# The Late Flood in the Ohio River

**Published:** 1848-01

**Authors:** 


### THE WESTERN JOURNAL.

THIRD SERIES.—VOL. I.—NO. I.

LOUISVILLE, JANUARY 1, 1848.

### THE LATE FLOOD IN THE OHIO RIVER.

On the 19th ult. (December) the late flood reached its maximum at Louisville, which was but eight or ten inches below that of February, 1832. It was at its greatest height, on the 17th, at Cincinnati, having come within four or six inches of the deluge of the year just mentioned. It appears from the newspapers, that this great altitude was reached at all the towns above, as far as Marietta; beyond which, it was not extraordinary, the maximum at Wheeling having been eleven, and at Pittsburg, eight feet below that of 1832. Referring to the same newspaper authorities, we find, on the south side of the Ohio, that the Monongahela, was not very high; but that the great Kenawha, Guyandotte, Big Sandy, Licking, Green, Cumberland, and Tennessee rivers, were greatly swollen; many of them, indeed, beyond all precedent. Passing to the north side of the Ohio, we perceive that the Alleghany was not remarkably full, but the Muskingum rose three feet or more above the flood of 1832; and the Sciota attained to a great elevation; the Little and Big Miamies, however, fell short of many previous freshets; and the general silence of the newspapers concerning the Wabash, indicates that it did not attain any great elevation; a conclusion which is strengthened by the fact, that the Illinois was frozen over, and the Mississippi, at St. Louis, in its ordinary winter condition.

From these facts, we percieve, that the copious and long continued rains, which caused the flood, extended from the Alleghany mountains, into the western parts of Tennessee and Kentucky, and eastern parts of Indiana, being most copious on the skirts of the mountains. In length, we know this zone extended from the southern waters of the Tennessee river, to the sources of the Muskingum—equal to seven degrees of latitude, but we may learn hereafter, that one extremity rested on the Gulf of Mexico, and the other on the Gulf of St. Lawrence. Its axis was nearly north east and south west. In its early stages, this rain was accompanied, at Louisville, with thunder and lightning—in its latter it changed to snow, which continued to fall on a wet surface, till it became one of the deepest ever known in the State. But for the reduction of temperature, which thus turned the rain into snow over most of the rainy zone, the inundation would have been much deeper. The wind and clouds were from the south west.

The great flood in the lower part of the Missouri, and middle section of the Mississippi, in the summer of 1844, was produced by rains which fell in a zone running nearly from W. S. W. to E. N. E. The axis of that, would intersect the axis of the late flood, at an angle of 22°.30′ beyond the St. Lawrence.

It is, or rather was, well known, that according to Indian tradition, there was a remarkable swell in the Ohio river, not very long before the first settlement of its banks by the whites. From information collected in Cincinnati, at an early day, it is supposed to have equalled any which has subsequently occurred. Since the settlement of the country, we have had the following: 1st. In the month of March, 1773. 2d. In February, 1832, or thirty-nine years afterwards. 3d. In December, 1847, or nearly sixteen years subsequently. These freshets were so nearly the same in altitude, that they may be taken, in that respect, as identical; and as about seventy-five years have elapsed since the settlement of the country, we may not expect ever to have a higher rise. At the same time, the occurrence of the third, within sixteen years after the second, ought to suggest to those who inhabit the lower bottoms, that they may be visited more frequently hereafter than heretofore.

The floods of 1793 and 1832 were caused by rain and melted snow; that of 1847 by rain only; which explains why the Alleghany and Monongahela rivers did not rise as high, compared with the rivers below, as in 1832. They now sent out rain water only, then rain and snow water mixed.

The great Missouri flood did immense permanent injury, by drifting sand upon the bottoms; but all inundations along the Ohio river deposit silt and increase the fertility of the soil.

It will be observed, that the whole of the great freshets have occurred between the autumnal and vernal equinoxes in December, February, and March. In January the weather is too cold. In summer and autumn the ground is so dry and hot, that, between absorption and evaporation, a great flood is likely to be averted. It might, however, occur in April or May. As bearing on the production of that from which we have lately suffered, it may be remarked, that the autumn had been uncommonly wet, and that, therefore, but little of the water which fell soaked into the ground.

